# The Experience of Being Born: A Natural Context for Learning to Suckle

**DOI:** 10.1155/2012/129328

**Published:** 2012-09-26

**Authors:** Jeffrey R. Alberts, April E. Ronca

**Affiliations:** ^1^Department of Psychological and Brain Sciences, Indiana University, Bloomington, IN 47405, USA; ^2^Department of Obstetrics and Gynecology, Wake Forest School of Medicine, Winston Salem, NC 27157, USA; ^3^Department of Neurobiology & Anatomy, Wake Forest School of Medicine, Winston Salem, NC 27157, USA; ^4^Molecular Medicine and Translational Science, Wake Forest School of Medicine, Winston Salem, NC 27157, USA

## Abstract

Understanding the developmental origins of congenital capabilities such as sucking is fundamental knowledge that can contribute to improving the clinical management of early feeding and facilitate the onset of oral ingestion. We describe analyses in rats showing that sensory stimulation *in utero* and during birth establishes the newborn's sucking responses to maternal cues. We mimicked elements of labor and delivery (viz., compressions simulating labor contractions, stroking simulating postnatal maternal licking of the newborn, and postnatal thermal flux), and used them to induce postnatal respiration and nipple attachment in caesarian-delivered pups. We report herein new data showing that, by simulating a fetal rat's experience of being born, specific components of vaginal birth provide sufficient conditions for the odor learning that guides newborn's sucking responses. In contrast, the absence of *in utero* compressions was associated with poor sucking onset. Knowing how birth stimuli contribute to the first nipple attachment and constitute a context for learning to suckle is an important step toward better management of some early feeding problems. It can serve also as a foundation for understanding the challenges of facilitating sucking by babies born prematurely so that they do not experience the typical contingencies mediating onset of oral ingestion.

## 1. Introduction

The ability of a newborn baby, fresh out of the womb, to attach to the maternal nipple and begin sucking leads many to label the behavior as “innate.” Some extend this concept of innate behavior to include reflexes, denoting fixed action patterns organized as sensory-motor circuits in the brain stem or spinal cord [1, 2], as well as the rhythmic firing of “central pattern generators” (CPGs), also located in the central nervous system, that produce correlated neuromuscular sucking rhythms [3].

 The concept of “innate” behavior is controversial, to say the least [4, 5]. Criticisms abound because such behaviors, when carefully observed and measured are not fixed but are highly variable [6, 7]. Moreover, so-called “instinctive” behaviors, under the scrutiny of experimental analysis, prove to be based on various forms of prior experience and learning [8, 9]. Similarly, while the firing of CPGs may correlate with sucking rhythms, it has yet to be shown that such isolated elements actually combine with other discrete components to create the real behavior of the suckling newborn, behavior that adapts to the unpredictable, dynamic geometry of the mother's body, and behavior in real time and in real contexts [6].

*Congenital *is a more accurate and defensible term with which to denote a capability in behavior that is present at birth. In contrast to “innate” and “instinctive”, congenital is more obviously a *description* of status at birth than it is an *explanation* of its basis or origins [10, 11]. Explaining the developmental origins of a congenital capability such as nipple attachment and sucking by a newborn upon confronting for the first time its mother's external body in highly novel environment is a formidable challenge of clinical significance.

The experimental literature, based on studies with non-human animals, contains a wealth of information pertinent to a better understanding of the onset and development of oral feeding skills. This literature includes some impressive findings concerning the onset of nipple attachment and sucking by newborn rat pups.

 A brief description of the postnatal onset of sucking by rat pups will help frame the forthcoming presentation and analyses: rat pups are born after a gestation period of about three weeks. They are born as litter, averaging 10 pups (mothers have 12 nipples), blind, deaf, and furless, with limited strength and coordination. For about 6 hrs prior to parturition, labor contractions can be seen rippling vertically on the mother's abdomen or indenting her sides as she stretches her body. More than 100 labor contractions can be observed during a rat's labor [12]. As each pup emerges from the birth canal, encased in an amniotic sac with the umbilical cord and placenta trailing, the dam assists with licking and gentle tugging. The mother removes the sac by licking and nibbling, consumes the placenta and membranes, licks the pup some more, licks herself, and then repeats the sequence as the next pup emerges. Only after all the pups are thus delivered and the placentas are all consumed, does the rat dam turn her attention to the newborns, which she gathers into a clump in the nest and settles over them.

We have described and quantified the labor and delivery process in rats, noting the stimulation received by the pups during labor and throughout the birth process [12]. In the nest, with the dam hovering above the pups, the infants are active. They orient to the dam's ventrum, probe against her body and root along the ventrum until they orally grasp a nipple and suck. It is difficult to observe directly the natural sequence of events that lead to the rat pups' initial nipple attachments and sucking. When the dam settles on her newly born litter she is typically crouched above the pups within a nest that affords poor visibility. Fortunately, the newborn rat's behavior is robust and orderly; when placed under suspended artificial surfaces simulating various properties of the dam's ventrum, newborn rats show an organized repertoire of behaviors. They travel, wriggle, turn on their sides and upside down, ventroflex, probe the surface, and audibly bark, all in a state of heighted behavioral arousal [13]. Other studies have been conducted with the dam anesthetized and the pup's behavior thus isolated for analysis. Thus, it is known that olfactory cues present on the rat mother's nipples and ventrum are necessary and sufficient for newborn pups to locate and orally apprehend a nipple to suck. These odor cues can be removed by washing the nipples and surrounding body surfaces that eliminates suckling [14, 15]. Nipple attachment and suckling by newborns can be reinstated, however, by painting onto the dam's ventrum a distillate of the wash taken from the dam's body or by painting nipples with amniotic fluid or maternal saliva [15]. Other substances, both natural and atypical were tried, but no others were effective in reinstating nipple attachment [15].

Knowledge that amniotic fluid is a sufficient stimulus to elicit the newborn's first nipple attachment led to preliminary considerations of two, mutually exclusive possibilities. One was that the key olfactory stimulus is somehow predetermined and that the newborn is correspondingly and inherently prepared (“hard-wired”, so to speak) to detect and respond to the cue. The second possibility was that the perinate responds with nipple attachment and sucking to the amniotic odor stimulus because of its previous experience with amniotic fluid. Pedersen and Blass [16] translated these contrasting explanations into an experiment with newborn rats. They reasoned that if amniotic fluid is a behaviorally potent stimulus because the fetus experienced it previously, then if some other odor was similarly experienced, it should have the same behavioral potency as amniotic fluid.

They tested this bold hypothesis by adding a novel, lemon-like substance to the amniotic fluid, and then testing whether this chemical would rescue the newborn's ability to make its first attachment to the washed nipples of a mother rat. The previously validated experimental procedure [17] involved externalizing the uterine horns of a gestational day (GD) 20 dam and injecting a small quantity (0.2 mL) of a citral and saline solution through the transparent wall of the uterus into the amniotic fluid. The uteri were replaced in the dam's peritoneum, the laparotomy incision was closed, and gestation was completed without complications. Then, on GD 22, pups were delivered by caesarean section and placed immediately for one hr in a warmed nest where they were stroked with “a soft artist's brush” for 1 hr in the presence of the citral odor.

The test procedure involved presenting the caesarean-delivered pups with an anesthetized parturient rat dam (not the subject pups' mother). If the dam's nipples were washed, pups did not attach to nipples, but when citral was on the dam, the treated newborns attached to a nipple and sucked! The new odor elicited the sucking behavior but, remarkably, “natural” odors of an unwashed dam were not effective for the citral-treated perinates. The new odor had replaced the natural stimulus. They performed an additional experiment in which pups were exposed to citral (a) *in utero*, (b) immediately after birth with stroking, or (c) both *in utero *and with postnatal stroking. Only pups with the combined experiences attached to the washed, citral-scented nipples and not to the unwashed, normal nipples.

Pedersen and Blass' [16] study provided important new insights into the initial plasticity of the newborn rats' sucking, especially the specification of the cues that can activate and direct the behavior. Their findings created a host of new questions. It seems clear that the establishment of the olfactory control of sucking is determined by the experiences of the perinatal rat pup. But, *what are the essential experiences for establishing the newborn's sucking responses to maternal cues?*

We adapted elements of our previous investigations of the perinatal rats' sensory experiences in the uterine environment and of the birth process [12, 18–20] to demonstrate that specific components of maternal stimulation are sufficient conditions for the odor learning that establishes the newborn's sucking responses to maternal cues. The present paper is a review of some of this past research as well as a report of additional, previously unpublished data that, together, provide a new view of how the *experience of being born *creates a context for learning. That is, embedded in flow of events that constitute the birth process are forms and levels of stimulation that, together, create the contingencies for early, rapid learning in the fetus, as it becomes a newborn. We will show that this learning, though general in initial form, is expressed in the natural context of the mother's body as organized, adaptive, seemingly goal-directed behavior.

We will first review an analysis of rat maternal behavior during labor and delivery from which we derived a set of novel tools that enabled us to simulate the major components of vaginal birth. We will also review some of our evidence that fetal and neonatal rats (perinates) have sensory capabilities sufficient to experience the birth process, at least the elements that are needed for basic associative learning. Then, we will present data showing that the perinates' responses to simulations of the birth process (a) augment nipple attachment and sucking, (b) establish odor-guided responses to the mother, and (c) induce neural conditions that mediate state transitions between fetal and neonatal behavioral systems which can account for the activation and expression of the newborn's initial sucking behavior.

**Figure 1:**
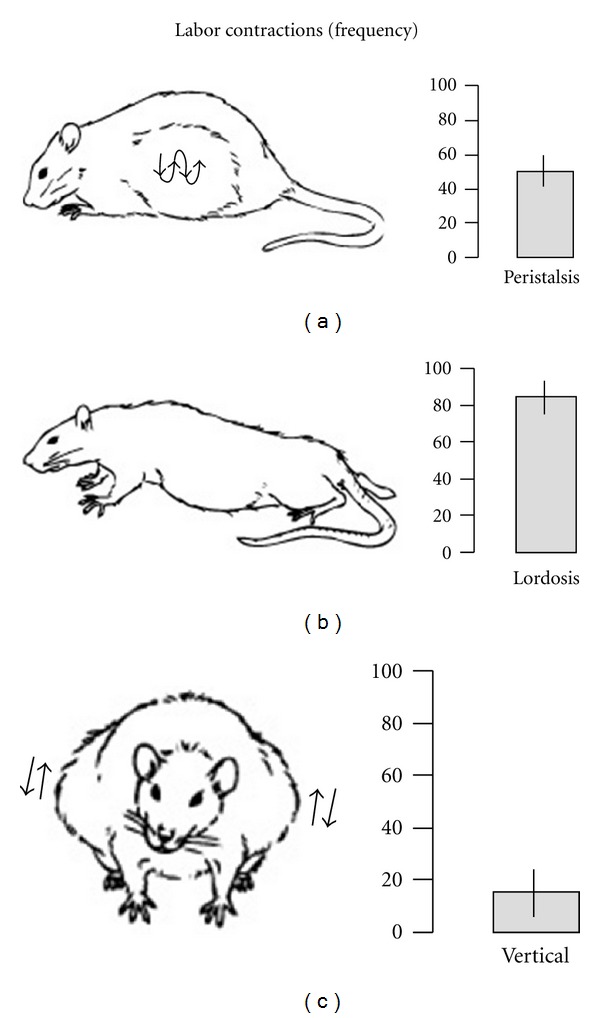
Frequency of three types of labor contractions observed in Norway rats. Histograms show mean frequency of peristalsis, lordosis and vertical contractions counted from videorecordings of labor in the six hours prior to delivery of the first pup (*n* = 8 dams). Based on data from [12].

**Figure 2:**
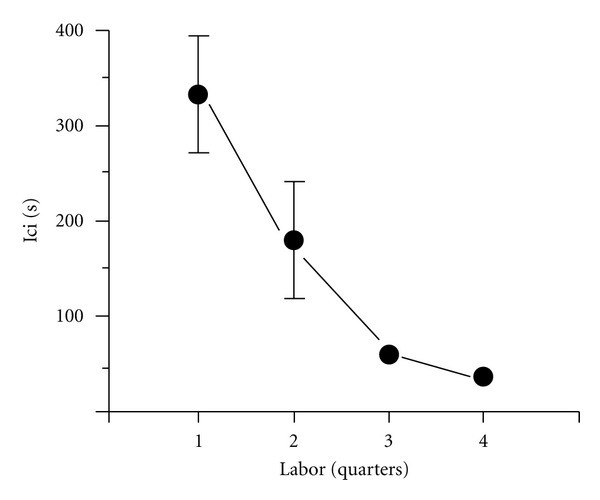
Intercontraction intervals (ici) during pre-birth labor recorded from rat dams (*n* = 8) from [12].

 Under laboratory conditions, Norway rats typically give birth on the 22nd day of gestation; by breeding our animals on a known day, we were able to be present with appropriate video arrangements to view and record the dams' labor and delivery [12]. From these videorecordings, we quantified an average of 144.6 labor contractions during the six hours prior to the birth of the first pup from eight dams.  Figure 1 illustrates the three types of visible labor contractions in rat dams and shows the average frequency of each during the 6 hrs of labor. Behavioral expressions of labor in the rat progress from uterine peristalsis to lordosis contractions followed by vertical contractions that occur in close association with birth of each pup. The brisk, linear decline in intercontraction intervals shown in Figure 2 indicates how the contractions quicken as parturition approaches.

From our systematic observations, we are able to describe the labor and delivery in the rat. Parturition in all 8 dams occurred within 12 hrs of E22. Duration of the delivery phase of parturition (first to last birth) ranged from 40 to 136 min, with dams delivering and average of 10.1 (±1.1) pups. Delivery duration and litter size were positively related (*r* ^2^ = .73, *P* < .01) across the dams.

As each pup begins to emerge from the birth canal, the dam typically adopts a head-between-heels posture, which facilitates delivery by enabling the mother to use her teeth to grasp the newborn and extract it from the vagina. Mothers lick and handle each pup, removing and consuming the embryonic membranes, activities that produce cutaneous stimulation and augmented evaporative cooling of the newborn's body. Dams provided about 2 min of continuous stimulation to each newborn while participating in its delivery.

Nearly all the pups were born in the vertex position. Thus, the initial stimulation from maternal licking was to the offspring's head. Each pup also received bursts of vestibular stimulation as the dam rotated its body while systematically consuming the products of gestation. During the initial phases of intense tactile and vestibular stimulation, pups were unresponsive. As birth membranes were removed, especially from the head, pups began to emit the robust gasps that are characteristic of the onset of independent respiration [21], and thereafter they displayed gross movements and audible vocalizations. After the immediate postpartum licking and handling of each newborn, the dam often refocused her attention on previous newborns, providing each one with about 2.5 min of additional licking and handling. Overall, licking by the dams was distributed relatively evenly across the pups' bodies: head (39%), body (24%), anogenital area (32%), (see [12] for additional details). 

Such observations help to specify events to which the newborn rat is exposed during birth. Each pup received a protracted bout of repetitive tactile and vestibular stimulation associated with the dam's handling and licking. During parturition, offspring are exposed to seemingly harsh forms of stimulation related to cooling and with compressions under the weight of the dam's body [12].

After systematically describing and analyzing the kinds and amounts of stimulation sustained by rat fetuses as they were being delivered vaginally, we endeavored to assess quantitatively some of the most prominent forms of stimulation. Among the forms of birth stimuli that we analyzed were uterine compressions, cooling and rewarming, and maternal licking. From these analyses, we created a set of procedures and tools to mimic the biological stimuli that represent the physical bases of the pups' experience of being born.

**Figure 3:**
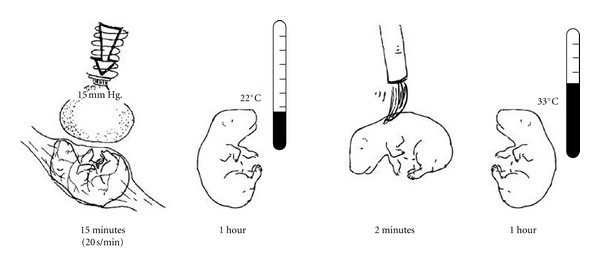
Four stages in a protocol for simulating a vaginal birth sequence. The drawings depict, from left to right: Simulated contractions delivered 15 times to a fetus still *in utero* but externalized from the dam's abdomen, once per minute for 20 sec each from a balloon attached to a calibrated (15 mm Hg) spring-loaded device; one hour of cooling after caesarian delivery, simulating a typical challenge faced by pups lying in the vicinity of their parturient dam still busy delivering other pups; two minutes of stroking with a soft brush, mimicking the postpartum licking by the dam; one hour of rewarming, simulating the heat received from the dam's body after being gathered and assembled in a nest with newborn littermates.

Uterine contractions, for example, were measured by surgically removing a single fetus from one of the paired uterine horns of a G18 rat installing a small balloon in its place *in utero*. The balloon was connected to a thin polyethylene tube that ran subcutaneously to the dam's back and was externalized at the nape of the neck [19]. The tubing could be connected to a pressure transducer with which we measured the forces exerted on the fetuses by the mother's behavior and by uterine contractions. The dams' contractions ranged from 2 to 30 mm Hg. By attaching an inflated balloon to a small, spring-based calibrated scale, we could apply with the balloon surface a reliable force of 15 mm Hg to a single fetal rat or to a newborn (see Figure 3). In this way, we established a protocol for simulating a vaginal birth for rat fetuses: 15, 20 sec-long compressions delivered at a rate of 1 per min, cooling (22°C), stroking with a soft brush (2 min), and rewarming (33°C).

Several of our analyses have focused on the how the birth process, beginning with the mother's labor contractions, helps organize the fetal-to-neonatal transition. Breathing and suckling are two vital behavioral adaptations of the newborn. In one set of studies, we applied our tools to study the components of birth that are important in the onset of pulmonary respiration, perhaps the most essential and immediate requirement of the newborn. The respiratory movements present *in utero* are episodic and unrelated to gas exchange [22, 23]. At birth, however, breathing becomes continuous and regulated to meet the newborn's oxygen requirements [24]. We found that compressions simulating uterine contractions were necessary for initiating breathing in late gestation rat fetuses. The effectiveness of simulated labor contractions could arise from some mechanical (nonsensory) effect of the compression, or cutaneous (sensory) effects on the offspring. In a study of gentle stroking of cesarean delivered pups (without simulated labor contractions), only 25% nonstroked pups survived for 1 hr postpartum compared to 100% stroked pups supporting a role for sensory stimulation. These observations fit well with reports of adaptive neuroendocrine changes and neurobehavioral advantages in neonates, both term and preterm, exposed to tactile and kinesthetic stimulation [25, 26].

We applied our “simulated birth” paradigm to examine more complex behavioral patterns in newborn rats in a study of how suckling becomes established [18]. Fetal rats were either exposed to labor contractions or not then cesarean delivered as described earlier, except that we manipulated postpartum ambient temperature using one of three biologically relevant temperatures. Newborns were exposed to the cool room-temperature environment (22°C) or to a warmer temperature maintained at nest (33°C) or intrauterine (36°C) temperature. After 1 hr postpartum exposure to one of the three temperature regimens, pups from all groups were placed at nest temperature then tested for nipple attachment. The 22°C condition contained the sequence of thermal exposures experienced by a vaginally born rat pup under typical thermal conditions. The treatment regime, then, was designed to represent the sequence and duration of stimulation that normally occurs prior to and immediately after vaginal birth, leading to the onset of suckling. At 2 hr postpartum, we found that 90% of vaginally delivered pups attached to a nipple (Table 1). For the cesarean newborns, both prenatal compression and postpartum temperature affected nipple attachment. The most dramatic effects of prenatal compression were seen between pups that experienced thermal conditions similar to those of normal, vaginally delivered pups (i.e., the room temperature condition), whereas thermal effects were most evident in pups exposed to atypically warm temperatures (i.e., the intrauterine temperature condition). Suckling was dramatically enhanced in compressed pups that underwent the naturalistic cooling episode.

These studies link the major postnatal milestones of pulmonary ventilation and suckling to birth experience. We sought to determine the mechanisms underlying the effectiveness of birth stimuli in facilitating the fetus-to-newborn transition. Human babies show a surge of plasma catecholamines associated with the “stress of being born,” a physiological response to labor and squeezing through the birth canal [27, 28]. Vaginally delivered infants show exhibit both enhanced respiratory performance and increased alertness compared to Cesarean-delivered infants whose mothers did not undergo full labor [29–31]. Catecholamine concentrations are higher in vaginally delivered human infants as compared to Cesarean-delivered infants [32]. We analyzed plasma catecholamines at 0 to 2 hr-old following either: (a) vaginal birth, (b) cesarean section with simulated labor contractions, or (c) cesarean section without labor contractions (mimicking planned cesarean delivery). Pups were exposed to the major elements of the rat's natural birth process, as we have described (i.e., umbilical cord occlusion, tactile stimulation and cooling). Only pups exposed to actual or simulated labor showed an immediate and profound rise in norepinephrine and epinephrine, to levels up to 35% greater than those of noncompressed pups. Our results, the first reported in the perinatal rat, closely parallel those reported in human studies and studies using the precocial sheep model [27, 33].

### 1.1. Catecholamine Release and Neonatal Adaptation to the Extrauterine Environment

Labor contractions do more than move the fetus through the birth canal. Whether by design (natural selection) or by incidental effect, contractions provide a form of stimulation that serves to facilitate two neonatal achievements: pulmonary respiration and suckling. Birth stimuli, that is, the range, levels, and patterns of stimulation that comprise the birth process, might have multiple roles in the successful transition from fetal to postnatal life [34]. Our simulated birth model incorporates actual forms and levels of sensory and physiological stimuli to which the rat is exposed during natural vaginal birth and allows us to specifically parcel out the effects of labor on postpartum functions.

The experience of labor is associated with a number of positive neonatal outcomes, including lung compliance, respiratory integrity [35–37], blood flow [38], resistance to oxidative stress [39], neonatal neurological condition [40], and complex global EEG patterns [41]. Human infants are particularly responsive to odors emanating from their mother's nipple/areola region and can identify the nipple by smell [42, 43]. Amniotic fluid and breast odors are regulators of infant sucking behavior, comfort, and distress reactions [44–46]. Learning about natural breast odors is enhanced in neonates that experience labor contractions, possibly mediated by NE [47]. Together with the results reported herein, these studies support the view that prenatal events associated with labor initiate a cascade of neural, physiological and behavioral changes that assist the neonate's successful transition to postnatal life events that assist the newborn infant's adaptation to the extrauterine world.

### 1.2. Experiment: Simulated Birth Experience Is Sufficient to Induce Odor-Guided Nipple Attachment

We now describe an original experiment conducted in our laboratory by Abel [48] in which individual, externalized, near-term rat fetuses received a combination of the simulated birth stimuli described earlier (see Figure 3) while in the presence of the odor citral and then tested for their responses to a rat dam with natural odors, washed of natural odors and with citral added. Specifically, while still residing in their amniotic sac and uterine horn that had been gently brought outside the dam's abdomen, each pup received a series of simulated labor contractions. Pups were next removed from the uterus, at which time there occurred a bout of tactile stimulation associated with removal of the birth membranes. Following this birth, each pup was stroked with a soft brush, mimicking the normal maternal licking and it also experienced cooling as it would after a natural birth and then rewarming as it would, had it been brought into the nest for maternal brooding. In effect, we created a simulated birth sequence. One set of pups experienced their birth in the presence of citral that was injected into the amniotic fluid prior to the intrauterine compressions and that was in the air around the pup while it was stroked and cooled and rewarmed. Alternatively, saline was used instead of an odorant for the littermate control subjects, that otherwise experienced the same birth sequence. The goal was to test the hypothesis that an arbitrary odor, paired with the experience of birth stimuli, would become a conditioned odor capable of evoking nipple attachment behavior from a newborn. The nipple attachment test was conducted with each rewarmed pup.

Our regime of stimulation was a controlled, 135 min analog of Pedersen and Blass' [16] 50-hr-long process used to induce a newborn rat's nipple attachment to novel odor. In contrast to their approach, we were able to specify and control the kinds, quantities, and timing of a specific stimulation sequence, and to observe the perinate at each stage of experimental manipulation.

We predicted that a perinatal sequence of experiences in association with an otherwise neutral olfactory cue would lead to rates of nipple attachment to that cue, similar to those of vaginally delivered newborn rat pups to the odor of amniotic fluid. If the outcome of the simulated birth experience was equivalent to a natural delivery, we would consider this a successful empirical demonstration of sufficiency. We will have demonstrated that the experience of a simulated birth, quantitatively comparable to a natural, vaginal birth, is sufficient to establish a conditioned response to an odor that is expressed as nipple attachment and the onset of sucking in an, otherwise, naïve newborn.

## 2. Methods

### 2.1. Subjects

Animal experimentation was conducted in accordance with the guidelines of the Indiana University Institutional Animal Care and Use Committee and the NRC Guide for the Care and Use of Laboratory Animals *(copyright 1996, National Academy of Science). *One hundred twenty-six fetal rats, derived from 24, time-mated Sprague-Dawley rat dams *(Rattus norvegicus) *were used as subjects. All breeding and maintenance was conducted in the Animal Behavior Laboratory at Indiana University. The first day that sperm was detected in a vaginal lavage was recorded as the day of conception (Gestational day [G]0), with birth expected on G22. Dams were group-housed in maternity tubs under standard vivarium conditions.

### 2.2. Treatment of Dams

On G21, pregnant dams were briefly anesthetized with isoflurane (Aerrane, Ohmed PPD Inc., Liberty Corner, NJ). An area overlying the lumbar region was shaved and a small (3 cm) dermal incision exposed the vertebral column. To eliminate movement and sensation below the ribcage, 100% ethyl alcohol (0.1 mL) was administered via intrathecal injection between the T12 and L1 vertebrae. After confirming loss of sensation, the female was placed in a Plexiglas holding apparatus and her lower body immersed into a heated (37.5°C ± 5°C) saline bath. A midline laparotomy was performed and the dams' paired uterine horns were gently externalized into the bath.

### 2.3. Treatment of Fetuses and Neonates

**Figure 4:**
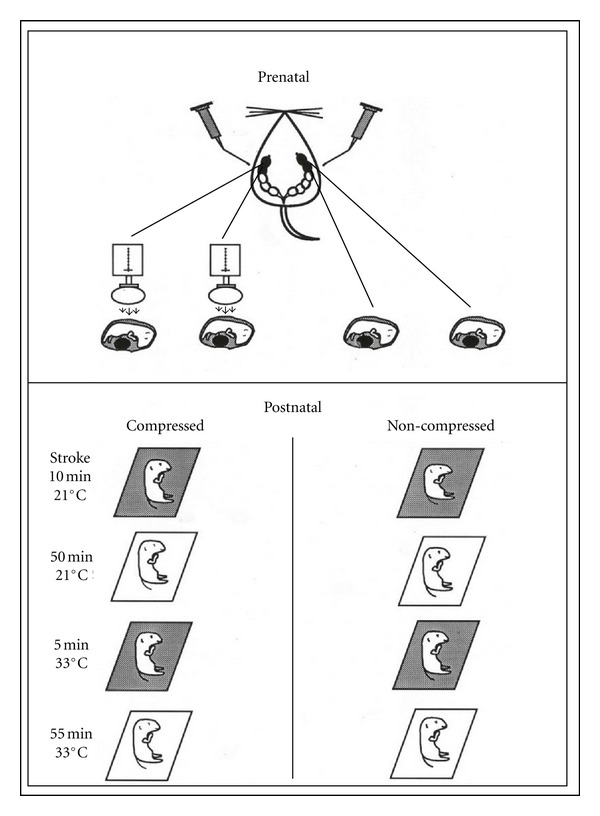
Sequence of perinatal manipulations prior to tests of respiration and nipple attachment. The top portion of the figure depicts injections of citral or saline into individual amniotic sacs, which was followed by compressions or no compressions (control). Pups were then delivered by caesarian section. Over the next two hours, individual pups were treated with thermal and odor regimes designed to mimic early postnatal events, shown in the lower sections of the figure. For some pups, citral was present prenatally and postnatally. Saline was used on the gauze pads of the control pups (see text). The grey and white areas around each pup depict the olfactory treatment (citral or saline control) of the moistened pads.

Figure 4 depicts the prenatal and postnatal manipulations. For each dam, either citral (Sigma Chemical Co., St. Louis, 50 *μ*L in 4 mL/L isotonic saline) or vehicle alone was injected into the amniotic fluid surrounding target fetuses. Beginning with the fetus in the second ovarian position, amniotic sacs of three-to-four adjacent fetuses were injected. A 30 ga hypodermic needle was inserted through the transparent uterine wall and into the amniotic sac near each fetus' snout.

Immediately following either citral or saline injections, compressions of 10–15 mm Hg pressure were administered to fetuses in one uterine horn using a small latex balloon. Such pressures are within the range of pressures typically experienced by rat fetuses during labor contractions [49–51]. Compressions were delivered at the rate of one, 15 sec compression per min for 15 min.

### 2.4. Cesarean Delivery Procedure

 Upon completion of the compressions, an incision was made along the antimesometrial border of each uterine horn. Fetuses were removed individually from the uterus and delivered onto gauze pads moistened with either citral (1 mL of 4 mL citral/L isotonic saline) or isotonic saline-moistened (1 mL) gauze pads (Figure 4).

 Immediately following delivery from the uterus, two cotton-tipped swabs were used to remove the birth membranes from the newborns, umbilical cords tied with surgical silk, and placentas removed. Each neonate was stroked with a soft-bristled artist's brush until respiratory activity was established (approximately 2-3 min per litter). Next, the newborns experienced temperature fluxes similar to those observed after a natural birth sequence in the laboratory [12]. They were placed onto saline-moistened gauze pads in individual glass dishes (Pyrex 80 × 40) at room temperature (22°C ± 0.5°C) for 60 min, then moved to an incubator maintained at nest temperature (33°C ± 1°C) for an additional 60 min. At 60 min postpartum, pups that had received pre- and postnatal exposure to citral were placed individually in glass dishes on citral-moistened gauze pads (1 mL citral solution/pad). These pups remained in the warm citral ambience for 5 min then transferred back to the original dishes containing saline pads in a noncitral incubator for the remainder of the second postnatal hr. Pups in both conditions were handled identically throughout the experiment except for the presence of citral. To ensure olfactory isolation, care was taken to maintain separate citral and noncitral incubators during the postnatal exposure and testing periods.

### 2.5. Postnatal Respiratory Behavior

Physical stimulation in the form of compression or stroking is necessary for the establishment of respiration in newborn rat pups [19, 20]. In the present experiments, postnatal stroking was required to elicit respiratory activity in the noncompressed subjects; equivalent durations of stroking were provided to all groups. To verify that alterations in frequency of suckling onset between compressed and noncompressed newborns were not related to deficits in respiration, respiratory movements were sampled (1 min) at 3 postpartum time points: 10 min postpartum; 1 hr postpartum (while at 22°C); 2 hr postpartum (while at 33°C).

### 2.6. Nipple Attachment Test

 Approximately 20 min prior to testing nipple attachment, a recently parturient (1-2 day postpartum) dam was anesthetized with a ketamine/xylazine mix (ip; 100 mg/mL; 0.9 mL/kg, 20 mg/mL; 0.5 mL/kg) and placed within a 33°C test incubator in the supine position. At 120 min post-delivery, each pup was gently grasped and held for up to 120 sec with its snout in contact with a nipple of the test dam [16]. Successful attachment to a nipple was verified visually and then by testing if the pup maintained oral grasp of the nipple while gently retracted from the dam.

 Following the first attachment trial, the dam was moved to a second heated (33°C) incubator and citral-scented gauze pads (5 pads; 1 mL citral solution/pad) were rubbed across the ventrum, thus infusing the fur with citral odor. Note that the solution was not placed directly on nipples. The scented pads were then placed alongside the dam, further contributing to the citral odor within the incubator. The nipple attachment test was repeated. Then, to further verify the effectiveness of perinatal exposure to citral in promoting nipple attachment to a citral-scented dam, one group of compressed but citral-naïve newborns was tested first on a citral-scented dam and then on a normal dam.

### 2.7. Statistical Analyses

 McNemar chi-square for dependent measures was used to analyze frequency of nipple attachment. A repeated measures ANOVA was performed on respiration scores. Posthoc comparisons were made with Newman-Keuls with a cutoff of *P* < 0.05.

## 3. Results and Discussion

**Figure 5:**
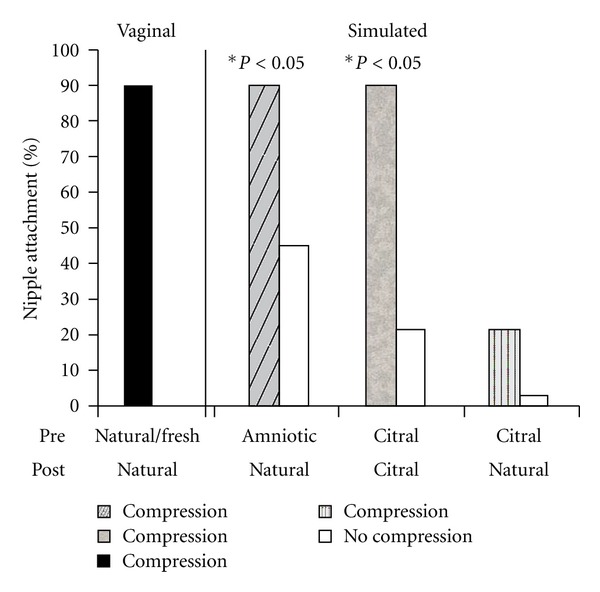
Percentage of pups attaching to a nipple of an anesthetized dam during a standardized suckling test. Shown below the horizontal axis are the prenatal olfactory conditions for each group and the lower row shows the odor conditions during postnatal treatments. The leftmost (black-filled) bar illustrates the baseline rate of attachment by vaginally delivered pups in these nipple attachment tests when the pups were exposed prenatally to natural amniotic odors and tested with a natural-scent dam (Vaginal group). The groups that received simulated birth experiences with different odors were also differentiated by the presence or exclusion of compressions. Within the set of Simulated birth groups, pups compressed in the presence of natural amniotic odors attached to natural-scented dams at rates comparable to the vaginally-delivered pups, but those that lacked compressions did not. If pups receiving compressions in the presence of citral (grey bar) also attached at the high rate to a citral-scented dam, but uncompressed pups in that group did not. The rightmost, striped histogram shows that pups receiving compressions in the presence of citral did not attach to natural-scented dams.

The nipple attachment test used in the present experiment reveals robust and reliable behavior in newborn rats. The leftmost histogram bar in Figure 5 shows that 90% of the vaginally-delivered newborns held before the ventrum of a natural (unwashed) anesthetized dam attached to a nipple and suckled. Note that this was the *first attachment* for each pup. Thus, this testing method enables rapid and reliable expression of the onset of postnatal ingestion, and the 90% attachment rate following vaginal birth can be used as standard against which we can evaluate the results of the simulated birth experiences.

 Newborn pups that experienced a simulated vaginal birth in the presence of natural amniotic odors, including the regime of *in utero *compressions—Caesarean delivery—membrane stripping—cooling—stroking—rewarming (see Figure 3) attached to a nipple in 89% of the tests, as shown by the first hatched bar in Figure 5. The legend under that bar, Amniotic Fluid/Natural, indicates that these pups experienced unadulterated amniotic fluid odors and were with a natural, unwashed dam. Newborns that experienced the a simulated vaginal birth *absent compressions *in the presence of natural amniotic odors, attached in only about 44% of the trials, which was a significant decrement in relation to littermates treated identically but with the compressions. The contrasting result is seen in the open bar next to the hatched bar in Figure 5. Thus, the complete simulated birth sequence (including compressions), produced rates of nipple attachment in newborns that were fully comparable to those in vaginally delivered pups.

 Newborn pups that experienced a simulated vaginal birth in the presence of citral in their amniotic fluid and in the atmosphere during stroking (Figure 4) attached to a nipple in 89% of the tests with a citral-scented dam, as shown by the stippled bar in Figure 5. Newborns that experienced the simulated vaginal birth *absent compressions *in the presence of natural amniotic odors, attached in only about 20% of the trials with the citral-scented dam, a significant decrement in relation to littermates treated identically but with compressions. The open bar, adjacent to the stippled bar in Figure 5, shows the contrasting outcome. Thus, the simulated birth sequence in the presence of citral, including compressions, produced rates of nipple attachment to a citral-scented dam that were fully comparable to those seen in vaginally-delivered pups and to pups that experienced the full simulated birth in the presence of amniotic odors when tested with a correspondingly natural-scented dam.

 The rightmost pair of histograms show that newborns that experienced simulated birth stimuli in the presence of citral in their amniotic fluid and in the atmosphere during stroking (Figure 4) when tested with a natural scent dam (no citral during the test) attached to a nipple in only 20% and 4% of the trials, for the compressed and noncompressed subjects. Clearly, newborns that experienced birth in a citral environment were not prepared to attach to nipples on the body of a dam with only the natural scent of the species. But, we know that these newborns are capable of attaching to a nipple, as evidenced by the performance of the simulated birth group depicted by the stippled bar. For the citral-birthed pups, citral had become a necessary stimulus for the initial attachment.

 Respiratory rates at the three, sampled time-points (10, 60, and 120 min) were unaffected by either citral or compression (*P* > .10). As expected, however, there was a significant increase in respiration within all groups during the final hour at the warmer temperature (*F*(2,120) = 320.8, *P* < .01).

 We see at least two broad findings in the results of this experiment. The absence of *in utero* compressions of the fetus was associated with poor performance in the onset of nipple attachment. It might be tempting to conclude that compressions mimicking labor contractions are necessary for efficient initiation of nipple attachment in the newborn, but the present experiment was not designed to allow such a conclusion. Pups in the Compressed and Noncompressed groups received other tactile and thermal experiences. Although we categorized each operation as a separate form of stimulation, the perinate might be less discriminating in its responsiveness and all forms of stimulation might simply be additive and incrementally increase the level of arousal in the pup. Thus, compressions might just add to the experience of general stimulation in the pup and nipple attachment rates might reflect levels of general arousal. Even if true, such an effect would not account for the second broad finding, that odors paired with birth stimuli become conditioned stimuli for nipple attachment. The experience of a vaginal birth, real or simulated, appears to give behavioral meaning to the odors experienced in association with the birth stimuli. Schaal and colleagues have suggested that amniotic odors provide a “bridge” from the fetus' prenatal world to its postnatal environment [52], and the present results suggest that this bridge is constructed by the experiences embedded in parturition and that they result in a newborn behavior that has been rapidly assembled to follow the bridge to a nipple and the onset of suckling.

Stimulation associated with labor and delivery plays a key role in assisting the fetus' transition to postnatal life by inducing and canalizing specific behaviors, and thereby operating as a critical link in the chain of behavioral adjustments required for adaptation to the postnatal habitat. Fetal sensory experience appears to set into motion physiological processes that permit the onset of postpartum behavior and the expression of early learning.

## 4. Conclusions and Reflections

It makes sense, both logically and scientifically, to discard the idea that suckling is an “innate” or “instinctive” or “hard-wired” behavior in a newborn baby. Nevertheless, it is also sensible to revel in the readiness and competence of a newborn mammal to adjust immediately to severance of its umbilical connection to the uterine world and to make an oral connection to the mother's body and begin suckling. We recognized the ability of a newborn mammal to suckle by designating it as a *congenital behavior*, that is, present at birth. Clearly, suckling is an important congenital behavior worth understanding—for it is one of the primary adaptations to newborn life for all infant mammals, serving not only nutritive, immunological, and general physiological functions, but it is also a powerful component of bonding with the mother and creating a social context which supports sensory, motor, and cognitive development.

Much of what we explored in the present paper are lines of research that have been important in demystifying the kinds of basic processes that can explain the newborn's congenital abilities to orient to novel features on the mother's body surface and to initiate the complex, but vital behavior of suckling. From the findings that we reviewed, it can be concluded that the combination of sensory and motor processes that constitute successful suckling are rapidly assembled during the course of perinatal events. We focused on the roles of birth stimuli and specifically on the *experience of being born. *In the experiments we described, rat pups that did not experience the mechanical and thermal forces associated with vaginal birth failed to make the fetus-to-newborn transition. Moreover, by providing individual perinates with a simulated birth experience, it was possible to induce in them the dramatic developmental changes that serve the transition from fetus to newborn.

 There is now abundant evidence that *learning* is an important component of the birth transition in rats and in humans. Thus, this perinatal learning takes place in the context of the experience of being born. It appears that the set of sensory, endocrine, and neural events that comprise the physiological transition of birth also serve as factors in the perinate's learning about the odor cues that are present *in utero* and carry over into the *ex utero* world. These are the same cues that the newborn then uses to orient to the mother's body and that stimulate nipple attachment and suckling. We are impressed by the multi-leveled functions of sensory and physiological events of birth, but much more remains to be understood about them and how they operate during parturition.

 Here, we can speculate on some of the implications we see when considering the experimental findings from rats in the context of human births and the onset of suckling. As students of mammalian development, we hold an inclusive view of the basic processes comprising reproduction and development. We tend to see commonalities across mammalian species in these realms. Indeed, our past initial analyses of rat parturition (e.g., [12, 19, 20, 34, 51]) were shaped by Lagercrantz and Slotkin's [28] perspective on the “stress” of human vaginal birth. The results of our experiments with rats resemble their observations with human birth, and we have been able to take advantage of opportunities to control and manipulate the birth stimuli to gain insights into the embedded and embodied learning processes. We are particularly struck by the contrasting picture presented by prematurely-born infants who enter the postnatal world at a stage of development when their sensorimotor function is not yet prepared for suckling. While it may be beautiful and exciting to witness the eventual onset of sucking in a baby born at less than 30 weeks of age, it is sobering to contemplate the dramatically different factors and unnatural schedule of experiences that direct the prematurely-born baby to suck: a premie's early postnatal development may be supported by intravenous nutrition and then gastric intubation until the baby presents signs of “readiness” for oral feeding. Nurses, therapists and parents may then use a variety of techniques to gently and gradually facilitate the transition to sucking and ingestion. How different it is for the baby born at term, for whom the process is short, and in many regards, even intense and abrupt. Clearly, babies can achieve successful oral ingestion via different developmental paths. We see great potential in understanding the necessary and sufficient developmental steps as precursors to improved management and guidance of early ingestion.

 Other researchers have compared the development of feeding skills of term babies and prematurely born infants. Schaal and colleagues (cf., [52]), for example, have focused on the absence of pairings of chemosensory cues and nutritive intake when babies are fed by gavage. Their perspective extends to many aspects of experience that normally contribute to the integration of breathing, suckling, and swallowing. Recognition of such differences may be an important step towards identifying factors that contribute to the problems experienced by some babies and that lead to the higher incidence of feeding disorders in children born prematurely (cf., [53]).

## Figures and Tables

**Table 1:** Percentage of newborn pups attaching to nipples of anesthetized dams (*N* = 9 per condition). *P < .05

Group	% attached
Vaginal birth (22°C)	90
Cesarean birth	
Room (22°C)	
Compressed	89*
Noncompressed	44
Nest (33°C)	
Compressed	56
Noncompressed	67
Intrauterine (36°C)	
Compressed	89
Noncompressed	78
